# Consistent responses of the microbial community structure to organic farming along the middle and lower reaches of the Yangtze River

**DOI:** 10.1038/srep35046

**Published:** 2016-10-11

**Authors:** Wenhui Wang, Hui Wang, Youzhi Feng, Lei Wang, Xingji Xiao, Yunguan Xi, Xue Luo, Ruibo Sun, Xianfeng Ye, Yan Huang, Zhengguang Zhang, Zhongli Cui

**Affiliations:** 1 Key Laboratory of Agricultural Environmental Microbiology of Ministry of Agriculture, College of Life Sciences, Nanjing Agricultural University, Nanjing 210095, China; 2 Key Laboratory of Soil Environment and Pollution Remediation, Institute of Soil Science, Chinese Academy of Sciences, Nanjing 210008, China; 3 Nanjing Institute of Environmental Sciences, Ministry of Environmental Protection, Nanjing 210042, China; 4 Department of Plant Pathology, College of Plant Protection, Nanjing Agricultural University, Nanjing 210095, China

## Abstract

Soil microorganisms play a crucial role in the biogeochemical cycling of nutrient elements and maintaining soil health. We aimed to investigate the response of bacteria communities to organic farming over different crops (rice, tea and vegetable) along the middle and lower reaches of the Yangtze River of China. Compared with conventional farming, organic farming significantly increased soil nutrients, soil enzyme activities, and bacterial richness and diversity. A Venn diagram and principal component analysis revealed that the soils with 3 different crops under organic farming have more number and percent of shared OTUs (operational taxonomic units), and shared a highly similar microbial community structure. Under organic farming, several predominant guilds and major bacterial lineages (Rhizobiales, Thiotrichaceae, Micromonosporaceae, Desulfurellaceae and Myxococcales) contributing to nutrient (C, N, S and P) cycling were enriched, whereas the relative abundances of acid and alkali resistant microorganisms (Acidobacteriaceae and Sporolactobacillaceae) were increased under conventional farming practices. Our results indicated that, for all three crops, organic farming have a more stable microflora and the uniformity of the bacterial community structure. Organic agriculture significantly increased the abundance of some nutrition-related bacteria, while reducing some of the abundance of acid and alkali resistant bacteria.

Soil is the fundamental resource of an agricultural ecosystem. Overuse of agricultural chemicals, such as fertilizers and pesticides, in conventional agriculture and intensive human activities have caused serious soil degradation and accumulation of pesticides^1^. Organic farming systems are an alternative to conventional agriculture^2^ that minimize the impact of agricultural practices on soil quality and the environment^3^. Due to these advantages, the land area under organic agricultural management reached 43.1 million hectares by the end of 2013, with an annual increase of 14%. The productivity of organically farmed land was equivalent to that conventional farming but was subject to less chemical and energy inputs^2^, reduced nutrient losses^4^, and lower global warming potential^5^. Vegetable fields under organic production even produced yields equal to those under conventional production^6^,^7^. Evidence showed that organic farming provided more biodiversity than conventional farming^8^ with respect to birds, plants, butterflies, insects, invertebrates and microbes^9^,^10^,^11^,^12^.

Soil microorganisms play a crucial role in the biogeochemical cycling of nutrient elements and maintaining soil health^13^,^14^,^15^. Farming practices have been proven to influence the composition of soil bacterial communities. Agricultural management had complex and diverse effects on the soil microbiome^16^,^17^. Arriving at universally valid conclusions about organic and conventional farming systems is difficult because of the complexity of the soil microbial community and the limitations imposed by the resolution of analytical methods. Organic farming has influences on domain-specific biomass^18^, increases richness^2^, reduces dispersion^19^ and shifts the structure of the soil microbiota^2^,^19^. Fertilizer was considered to be the primary driving factor leading to the changes in soil microbiota. A significant difference was detected in the composition of bacterial genera between organic and conventional management systems^20^. Organically managed soils exhibit greater biological activity than conventionally managed soils^2^,^21^.

Community structure is essential for the ecological function of soil microbiota. The change in soil microbial diversity had a great impact on the stability of the soil ecosystem^22^,^23^; the relationships between the diversity of the microbial community and the function and stability of the community are very complex. Organic farming lands showed a tendency to suppress plant pathogens. Foliar diseases, such as stripe rust, powdery mildew, and snow mold of wheat, as well as soil-borne pathogens, were observed to be less severe in organic fields than in conventional fields^24^. An analysis of the fungal community by 454 pyrosequencing showed that organic farms had a slightly higher diversity and evenness with respect to the microbial community compared with conventional farms. The relative abundance of some potato fungal pathogens was less in organic farms^25^. *Aureobasidium pullulans* was considered to contribute greatly to the control of pathogenic fungi and to the better taste of organically produced wine^26^. Plant growth-promoting bacteria belonging to the *Burkholeria*, *Stenotrophomonas* and *Pseudomonas* genera were also more abundant in an organic farming system^20^. However, contrasting results were also observed in a six-year-long organic cropping study^27^. Characterization of the specific microbes involved in soil nutrient cycling or soil disease suppression that are significantly impacted by organic farming is necessary. Such microbes may be used to as indicators to monitor soil health. Although much work has been carried out to evaluate the impact of organic farming practices on soil microbiota, many farms containing different crop species and in various climate zones should be analyzed to draw clear conclusions regarding the differences between organic and conventional farms^25^.

Cases studies showed that many factors were positively respond to the organic farming. Such as soil enzyme activity^2^,^28^, soil nutrients^2^,^4^, diversity of animals and plants^9^,^10^,^11^,^12^, and microbial abundance^8^. Meanwhile, all these indicators are directly or indirectly related to soil microorganisms. That raises a series of questions: Will microbial communities show similarly positive responses to organic farming under different crops plantation? What are the main microorganisms involved in responding to these positive changes? What ecological functions do these changed microorganisms possess? Organic farming developed rapidly in China and became disseminated around the country due to its food and environmental safety a. In this study, we cooperated with the Organic Food Development and Certification Center of China (OFDC), to investigate the relationship between the soil microbial community structure and organic farming. We selected 12 organic and conventional crop production systems, which are mainly distributed in central and eastern China, to explore the responses of the microbial community structure to organic farming.

## Results

### Soil physical and chemical indexes and soil enzyme activities

Among organic farming systems of different ages, compared with the conventional farms, the soil physical and chemical indexes of organic farms were significantly changed (Table S2). The ANOVA results (Table 1) show that (1) there were significant differences in pH (soil pH), OM (organic matter), TP (total P), VP (available P), NO_3_-N (soil nitrate nitrogen), NH_4_-N (ammonium nitrogen), TK (soil total K) and VK (available K) between conventional and organic farms (P < 0.01); However these soil physical-chemical factors were not significantly different among different crop types. There existed significantly (P < 0.01) interaction between production system and location, in pH, OM, TP, VP, NO_3_-N, NH_4_-N, TK and VK. Soil pH was slightly higher in the organic groups (Table S2). Even if different natural conditions exist in different regions, the organic group had higher (P < 0.05) content OM, TP, VP, TK and VK contents than the conventional group, with the exception of NO_3_-N, NH_4_-N and SBD (soil bulk density); These results showed that organic farms can maintain or even increase soil fertility more effectively, as evidenced by factors such as OM, TP, VP, TK and VK. More interestingly, we found no significant difference in TN (soil total N) between organic and conventional farms. However, the available nitrogen (NO_3_-N and NH_4_-N) in soils under organic management was significantly lower than that in soils under conventional management.

**Figure 1:**
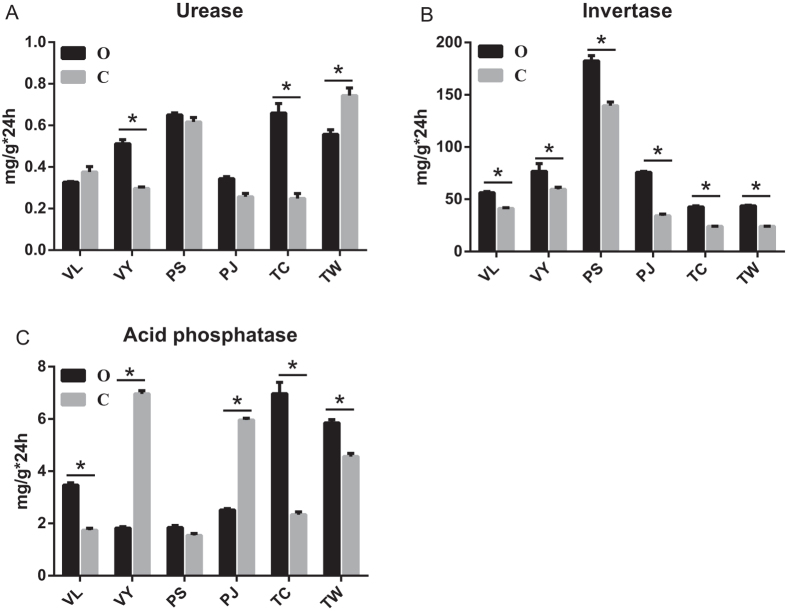
Soil enzyme activity in the 12 fields (P < 0.05, average value, n = 3). “O” represents organic farming; “C” represents conventional farming; “V” represents vegetable; “P” represents paddy; “T” represents tea; the last letter represents experimental region (the first letter of the sampling area).

The enzyme activities in relation to the cycling of carbon (C), nitrogen (N) and phosphorus (P) were determined by the methods of Zhou *et al*. (Zhou^73^). Soil invertase, urease, and acid phosphatase activities in the OTW and OTC sites were significantly higher than in the corresponding CTW and CTC sites (Fig. 1). Soil invertase activity in the organic fields was significantly higher than that in the conventional field, but the soil urease enzyme activity was not significantly different between the OVL and CVL, OPJ and CPJ, OPS and CPS sites. Acid phosphatase enzyme activity in OVL, OTW and OTC was significantly higher than that in the corresponding conventional sites (CVL, CTW and CTC) (P < 0.05); However, this activity in the OPJ and OVY sites, which had a partial neutral pH, was lower compared with the conventional sites.

### Richness and diversity of microbial communities

A total of 2,401,529 high-quality 16S_V4–V5_ sequences were obtained from all 36 samples were, with an average of 66,709 sequences (varying from 52,052 to 79,300) per sample (Table S3). A total of 10,865 OTUs were obtained, with 97% sequence similarity. Rarefaction curves showed that the paddy and vegetable soils had the steepest rarefaction curves with the highest taxon richness, whereas the tea soil had the lowest curves (Fig. S1). Organic farming had higher OTUs numbers than conventional farming, especially for tea soil.

Bacterial community richness was estimated with different richness estimators including Chao1 and the Abundance based Coverage Estimator (ACE). The results of Chao1 and ACE (Table 2) indicated that organic farming systems had greater microbial richness. Organic farming management had a significant positive effect on OVY, OTC, OTW and OPJ, whereas the vegetable soil of Lishui and the paddy soil of Shanghai were unaffected. The tea soils had lower richness (Chao 1 and ACE) and were more significantly affected by organic farming than the other two soils. According to Shannon and Simpson (Table 2), the organic group (except OPS) had a higher diversity, with the Shannon index ranging from 5.98 to 6.76, whereas the conventional group (except CPS) had a lower Shannon index, ranging from 4.91 to 6.11, and significant differences (P < 0.05) were evident between the two groups (Table 2). Similarly, the diversity in the tea, vegetable and paddy soils of Jurong was significantly higher under organic farming than under conventional farming, whereas no differences were found between organic OPS and conventional CPS.

### Bacterial community structure

**Figure 2:**
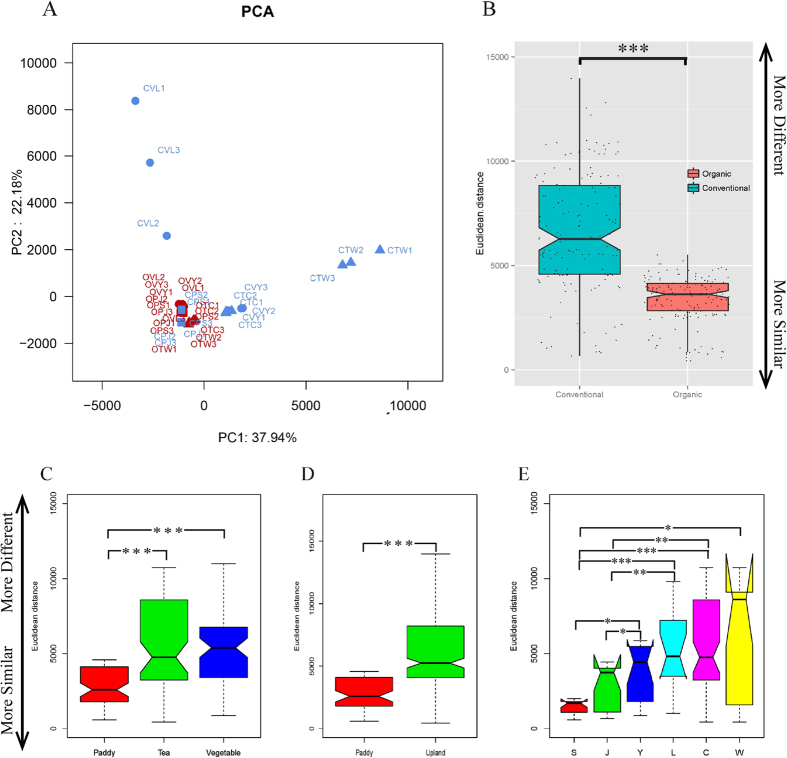
(**A**) PCA based on total OTUs level information, PC1 and PC2 were used to plot the result. The first letter of the field name represents the farming mode; “O” represents organic farming; “C” represents conventional farming; the second letter represents the crop type; “V” represents vegetable; “P” represents paddy; “T” represent tea; the third letter represents the experimental region (the first letter of the sampling area). (**B**) Similarity between organic and conventional samples. Euclidean distance between soil pairs is shown. (**C**) The Euclidean distance values of paddy, tea and vegetable soils. (**D**) The Euclidean distance values of paddy and upland soils. (**E**) The Euclidean distance values of S, J, L, Y, C and W. The nonparametric Wilcoxon test was used to calculate significance among the different sample groups (*P < 0.05; **P < 0.01; ***P < 0.001; ns, not significant).

Principal component analysis (PCA) (Fig. 2A) was used to compare the similarity of the soil bacterial community among the samples. PCA was performed to compare organic and conventional management at the OTU level. PCA identified two principal component factors (PCF) in relation to the percentage abundance of groups, explaining 37.92% and 22.18% of the total variation. PCA showed that the two types of management could be separated and that the organic farming samples were inclined to cluster together with high similarity, whereas the conventional samples were more decentralized. These results suggested that the microbial community structures were significantly modified by organic farming. Under conventional management, the bacterial community had marked diversity among the different crop fields, whereas under organic management, the bacterial community had fewer differences among the 3 crops. Organic farming caused the soil bacterial community to be more consistent, although for a different type of crop. When crop type was considered individually, there was higher similarity among samples of paddy soil other than among samples of vegetable soil and tea soil, which indicates that vegetable soil and tea soil were more sensitive to the different management practices than paddy soil.

**Figure 3:**
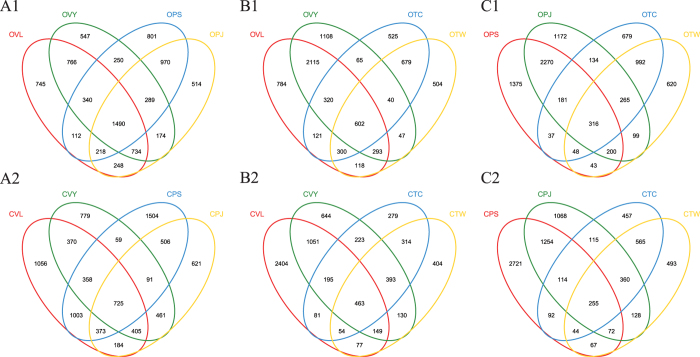
Venn diagram showing the unique and shared OTUs between organic and conventional soils. OTUs defined at 97% sequence similarity. (**A**) Venn diagram for vegetable and paddy soil; (**B**) Venn diagram for vegetable and tea soil; (**C**) Venn diagram for paddy and tea soil.

To compare community structure and similarity between organic and conventional samples, we determined the Euclidean distance between soil pairs. The Euclidean distance between organic pairs was significantly lower than that for conventional samples (Fig. 2B), supporting the observation that organic farming provided more similar bacterial community structure. At the same time, we also conducted the difference significance test on other kind of grouping (Fig. 2C–E). Different crops and water management practices (paddy soil and upland soils) exert influence on bacterial community. But organic or conventional management were still the important factor that influence the bacterial community. The shared OTUs for different crop types, locations and management models were determined via the Venn diagram (Fig. 3). For the vegetable and paddy soil, a total of 1490 OTUs (18.18%) could be detected in all four organic soils and 2607 OTUs (745 + 547 + 801 + 514; 31.76%) were unique to the soils they were found in (Fig. 3A1). In contrast, the shared OTUs were 609 (8.53%) only found in the conventional soils, and unique OTUs were 3501 OTUs (1056 + 779 + 1504 + 621; 41.32%) (Fig. 3A2). A similar pattern was observed for the vegetable and tea soil, in organic group, the number of shared and unique OTUs were 602 OTUs (7.90%) and 2921 OTUs (784 + 1108 + 525 + 504; 38.33%) (Fig. 3B1). But 463 OTUs (6.75%) and 3731 OTUs (2404 + 644 + 279 + 404; 54.38%) in conventional group (Fig. 3B2). Similar to all above results, for the paddy and tea soil, the percentage of shared and unique OTUs in the organic soils accounted for 316 and 3828 OTUs, respectively (Fig. 3C1). But 255 and 4739 OTUs in the pair of conventional (Fig. 3C2). All these result showed that the organic soil have more number and percent of shared OTUs, but lower number of unique OTUs.

For each crop type, we used redundancy analysis (RDA) to discern the OTU-level structure using the environmental variables (Fig. S2). The paddy, vegetable and tea soil samples were grouped according to the management system (O and C). The O and C management systems were differentiated along the first and second (or third for paddy soil) axes. These results suggest that pH, TP, TK and VK were key factors in shaping the microbial community functional structures in this system.

### LEfSe analysis based on community abundance

*Linear discriminate analysis (LDA) effect size (LEfSe)* is an effective statistical tool for high-dimensional biomarker discovery and the explanation of the detailed identification of abundant features that characterize potential discriminating taxa between two or more biological groups^29^. LEfSe was used to evaluate the bacterial groups that were significantly different between the two management models. All 36 samples could be separated into organic and conventional groups. We chose LDA scores higher than 2 to identify bacterial groups with statistically significant differences (see Table S4 in the supplemental material).

**Figure 4:**
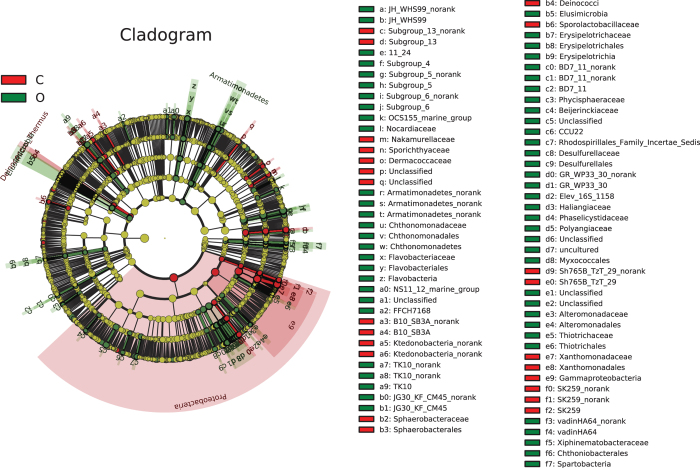
LEfSe cladogram of comparison result between organic and conventional samples. The black circles from inner to outer stand for phylum, class, order, family, genus, and species. Green circles stand for taxa which were abundant in the organic group and red circles stand for taxa which were abundant in the conventional group.

The cladograms show taxa with LDA values higher than 2.5 for clarity (Fig. 4). Some major groups of bacteria were enriched in the organic group, namely, Flavobacteria. Actinomycetales (the order and family of Micromonosporaceae, Pseudonocardiaceae, and Streptosporangiaceae), Chthonomonadetes, Phycisphaerales, Spartobacteria, Erysipelotrichia, Rhizobiales (the order and families of Beijerinckiaceae, Hyphomicrobiaceae, Xanthobacteraceae, Rhizobiaceae, Phyllobacteriaceae and Bradyrhizobiaceae), Novosphingobium, Rhodospirillales, Myxococcales (the order and family of Phaselicystidaceae, Polyangiaceae, Nannocystineae, and Myxococcaceae), Desulfurellales, Thiotrichales, Alteromonadales, Cupriavidus. Within these groups, 5 fine lineages had an LDA value of 3 or higher, namely, *Myxococcales*, *Planctomyces*, *Phycisphaera*, *Prosthecomicrobium* and *Lacibacter*.

The bacterial lineages enriched in the conventional soils were *Proteobacteria*, *Gammaproteobacteria*, *Xanthomonadales*, three groups in Actinomycetales (Microbacteriaceae, Dermacoccaceae and Nakamurellaceae), Ottowia, Ruminococcaceae, three groups in Clostridi (Sporobacter, Alicyclobacillu and Alkaliphilus), Ktedonobacteria, Sphaerobacterales, Sporolactobacillaceae, Acidiphilium, and Terriglobus. Only Gammaproteobacteria, Xanthomonadales, Proteobacteria and Xanthomonadaceae had LDA values higher than 4 in the conventional samples (Fig. 4).

## Discussion

Organic farming has become one of the most popular sustainable strategies to produce agricultural products^8^. Compared with conventional farming, organic farming was purported to improve the soil ecosystem quality^2^,^5^. However, solid scientific evidence is needed to clarify the ecological effects of organic farming. Soil bacteria are important member of the soil ecosystem and indices of soil health^30^. Elucidation of the soil bacterial structure will shed light on the regulatory pattern of soil microbial population^8^,^31^.

Farm management directly affected the nutrient input^2^,^4^, and microbial community changed in response to the soil nutrients^32^. Organic management significantly raised the pH, contents of OM, TP, VP, VK and TK of the organic soils investigated in this research. Meanwhile, bacterial richness, bacterial diversity, and soil enzymes’ activity (invertase, urease, and acid phosphatase) also increased comparing to conventional farming. These results implied the linkage between soil management, soil nutrients and the soil microbial community. Microbial community fed back to soil nutrients by modifying soil enzyme activity^2^,^28^.

Our results showed that organic farming led to more richness and diversity than conventional farming. Recent studies show that rejuvenation of ecosystem function requires the restoration of species evenness, rather than richness alone^33^. Organic farming potentially offers a means of recovering functional evenness in an ecosystem^33^. A number of studies have confirmed that soil microbes are often more diverse and abundant under organic than conventional systems in various soils^33^,^34^,^35^,^36^. The abundance of some soil microbial groups was increased under an organic system.

PCA demonstrated that the two types of management could be separated at the OTU level (97% *ID*); the organic samples tended to cluster together with high similarity, whereas the conventional samples were more decentralized. Sequences sharing a 97% identity or higher are normally defined as a bacterial “species” although no rigorous species concept for bacteria exists^37^. The clustering together tendency was also observed in archived soils^38^. A comparative study of organic and conventional commercial olive farming systems, discriminant and NMDS analyses proved especially effective in differentiating the 93 olive orchards according to their management system (conventional, organic or wild olives)^39^. 14 olive orchards from Córdoba and Granada provinces in southern Spain could be distinguished according to their management (conventional, organic or integrated)^40^. These organic soils showed in general greater soil nutrients, enzyme activities and showed intermediate characteristics (such as pH and OM) which may be attributed to the fact that organic farming management led to a similar microbial community structure. In addition, there were similar agronomic management practices as those in organic farms (i.e., out of use of Pesticide, use of cover crops, animal grazing or light tillage to control weeds or incorporation of animal manure as fertilizer). Under organic farm, the organic manure incorporation may have fostered the bacterial pathway of the soil food web^2^. Organic amendments had a positive impact on soil microbiology, as suggested by the increased amount of microbial C, N and P^41^. The bacteria Co-correlation networks were strongly bimodal response to farmyard manure application^42^.

LEfSe was used to evaluate bacterial groups that showed a significant difference between the soils. In the present study, the results showed that different management practices had a significant effect on the soil microbial community. As shown in Fig. 4, the organic group evidenced multiple shifts of specific bacterial lineages distributed in a multitude of lineages, and most of these bacterial lineages were involved in nutrient cycling. For example, members of the genus *Flavobacterium* are responsible for heterotrophic denitrification^43^. Recent studies show that *Verrucomicrobia* are ubiquitous in soils and appear to be predominant in many soil bacterial communities^44^, which are predominantly comprised of the Class *Spartobacteria*^44^,^45^. Although we know very little about *Verrucomicrobia*, they are generally considered to be free-living disintegrators. The *Sphingomonadaceae* family has the ability to utilize a great diversity of C sources, including recalcitrant xenobiotic molecules^46^,^47^. *Micromonosporaceae* (a family within *Actinomycetales*) are associated with secondary metabolite production, and some species are efficient solubilizers of rock phosphate^48^. Some reports note that *Micromonosporas* can degrade complex recalcitrant materials such as cellulose and chitin^49^,^50^, and produce acid and alkaline phosphatases^51^. It is interesting to note that our results found multiple genera of *Rhizobiales* and *Myxococcales*. *Rhizobiaceae* (*Sinorhizobium*) is involved in N cycling^52^; *Bradyrhizobiaceae* (*Rhodopseudomonas*), *Xanthobacteraceae* (*Starkeya*) and the *Hyphomicrobiaceae* (*Rhodoplanes*) are mainly involved in C and N cycling. Under hypoxic conditions, the genera *Bradyrhizobiaceae* and *Hyphomicrobiaceae* can also utilize N_2_, NO_3_^−^, or NH_3_^53^,^54^,^55^. Members of the *Myxobacterial* community act as micropredators that are metabolically active in the soil microbial food web^56^, and play a vital role in the turnover of carbon in soil ecosystems^57^,^58^. *Alteromonadaceae* is generally found in coastal environments, and have a potential activity in crude oil degradation^59^. The families *Thiotrichaceae* and *Desulfurellaceae* are mainly involved in S cycling^60^. In this study, invertase, urease, and acid phosphatase activities increased under organic farming, indicating that a changes in bacterial communities increased soil nutrient cycling.

In comparison, the relative abundances of *Gammaproteobacteria*, *Xanthomonadales*, *Ruminococcaceae*, *Sphaerobacterales*, *Acidiphilium*, *Acidobacteriaceae*, *Sporolactobacillaceae*, *Alicyclobacillu* and *Alkaliphilus* were significantly lower in organic than in conventional soil. The genus *Xanthomonadales* has anti-microbial activity against competing rhizosphere microorganisms^30^. A greater abundance of acid and alkali resistant microorganisms (*Acidiphilium*, *Acidobacteriaceae*, *Sporolactobacillaceae*, *Alicyclobacillu* and *Alkaliphilus*) were found under conventional tillage, suggesting that conventional tillage may exert stress on soil microbiota and that organic tillage is an eco-friendly pattern that encourages sustainable development.

A shift in particular bacterial lineages caused by organic management could be used as a bioindicator to assess soil quality in an agricultural production system, or used for the production of bio-organic fertilizer. For example, *B. asahii* originated from an archived OM fertilized soil sample^61^, which played a key role in increases in both crop yield and soil fertility, especially by accelerating carbon and phosphorus cycling.

Organic management of soil is generally considered to enhance soil disease suppression. This phenomenon has been found in many crops, such as tomato^62^, wheat^63^, barley^64^,^65^ and apple trees^66^. Various soil physicochemical factors and some disease-suppressing bacterial taxa can contribute to disease suppression^30^,^67^. Disease- suppressive bacteria were reported to play key roles in soil suppression. *Firmicutes*, and *Actinobacteria*, which were enriched in organic farming, were consistently associated with disease suppression^30^. In addition, certain bacterial genera, such as *Pseudomonas*, *Bacillus*, *Burkholderia*, and *Actinomycetes*, were often found in high populations in soils that were disease suppressive^67^. It is interesting that *Myxococcales* (*Cystobacteraceae* and *Haliangiaceae*), which were enriched on organic farms, have the capacity to produce secondary metabolites that have antibacterial and antifungal activities, such as Althiomycin and Myxovirescin^68^,^69^,^70^. A previous work showed that myxobacteria effectively kill microbial pathogens^71^, and exert partial control of damping-off and root disease in container-grown tree seedlings^72^. Populations of myxobacteria were stable. Zhou^56^ retrieved 103 pyrosequencing datasets in various soil samples from the MG-RAST and SRA databases to determine the abundance of myxobacteria. Their result showed that myxobacteria were among the predominant bacterial groups, accounting for 0.40–4.50% of the bacterial sequences. In our 36 soils, myxobacteria accounted for 0.25–4.02% of the bacterial sequences. We suspected that myxobacteria was a potential disease-suppressing bacteria, and played a key role in organic farming. However, further research is needed.

Miseq sequencing technique was used to analyze the consistent responses of bacterial community to organic farming for its ability of providing huge amount of microbial information. Our results showed that organic farming tends to maintain a highly similar microbial community structures regardless of crop types planted or soil management practices and soil types. Organic farming leads to a more stable microflora, and is beneficial to soil health and sustainable use of soil. Organic agriculture significantly increased the abundance of nutrition-related bacteria such as Rhizobiales, Thiotrichaceae, Micromonosporaceae, Desulfurellaceae and Myxococcales, and reduced the abundance of acid and alkali resistant bacteria.

In conclusion, organic farming significantly improved soil nutrient status and soil enzyme activity and enhanced the richness and diversity of the soil bacterial community. The microbial community in organic soils tended to converge at the OTU level. Dominant guilds involved in nutrient (C, N, S and P) cycling were significantly enriched, leading to new dynamics between the C, N, P and S cycles. Soil pH and soil nutrients were found to be nearly equally important in influence the microbial community, and the composition of the soil microbial community was strongly correlated with soil nutrients such as TP, VP, NH_4_-N, TK and VK in both organic and conventional systems. Organic farming promoted the uniformity of the bacterial community structure for all type of crops investigated. Compared with rice and vegetable crop soils from east China, tea soils from central China were more responsive to organic farming. All of these results allow us to better understand the differences between organic and conventional farming for different crop systems and the linkage between soil management, soil nutrients and the soil microbial community.

## Materials and Methods

### Study area

Rice, vegetable and tea are the most common crops in eastern and central China and are widely grown in this region for the conventional and organic markets. We relied on the OFDC to investigate the effects of organic management on microbial communities in agricultural soil and used conventional farming as a control. We finally selected 6 organic crop production systems (Certified organic by OFDC) and 6 corresponding conventional crop production systems. The vegetable soils were collected at Lishui [labeled as OVL (119°02′E, 31°39′N) and CVL (118°58′E, 31°36′N)], and Yangzhou [labeled as OVY (119°8′E, 32°25′N) and CVY (119°8′E, 32°22′N)] in Jiangsu Province, in China. The paddy soils were collected at Shanghai [labeled as OPS (121°12′E, 31°48′N) and CPS (121°12′E, 31°45′N)], and Jurong [labeled as OPJ (119°13′E, 31°39′N) and CPJ (119°13′E, 31°41′N)], in Jiangsu Province, in China. The tea soils were collected at Wuyuan [labeled as OTW (117°44′E, 29°26′N) and CTW (117°49′E, 29°20′N)], in Jiangxi Province and from Changsha [labeled as OTC (113°19′E, 28°33′N) and CTC (113°20′E, 28°34′N)], in Hunan Province, in China. Nutrient input and weed control varied among farms (Table S1), but these actual operational farms more accurately reflected actual microbial community information.

### Soil sampling and analysis

The sampling sites are shown in Table S1. The sampling spatial coordinates were recorded by GPS. Surface soil samples were collected in September 2013. In each field, three plots were established at random locations within a 0.25 ha area (50 m × 50 m). Soil samples were collected from each plot at 12 points at a depth of 0-20 cm, situated 15 cm from the centerline of the planting row, and then mixed and homogenized by passing through a < 2 mm sieve to remove aboveground roots, visible residue, and stones. The fresh soil samples were stored in polyethylene bags and subdivided into two subsamples; One was stored at 4 °C to determine its physical and chemical properties, and the other was stored at −20 °C prior to DNA extraction and microbial analysis.

Soil invertase, urease, and acid phosphatase activities were measured using the method of Zhou *et al*.^73^. Soil pH was determined with a glass electrode as previously described^74^. Soil total N (TN) and organic matter (OM) were determined by Kjeldahl digestion^75^ and determined by oil bath–K_2_CrO_7_ titration method^76^. Soil total K (TK) and total P (TP) were determined by digestion with HF-HClO_4_^77^, followed by flame photometry and molybdenum-blue colorimetry^78^, respectively. Available K (VK) was extracted by ammonium acetate and determined by flame photometry^79^. Available P (VP) was extracted by sodium bicarbonate and determined using the molybdenum blue method^80^. Soil nitrate nitrogen (NO_3_–N) and ammonium nitrogen (NH_4_–N) were extracted from 15 g of fresh soil with 2 M KCl (soil: extract/1:5) and analyzed using a continuous flow analytical system (San++ System, Skalar, Holland)^81^.

### DNA extraction and PCR amplification

DNA was extracted from the soil samples using an Ultra Clean microbial DNA isolation kit and from soil samples using the FastDNA® SPIN Kit for soil (MP Biomedicals, Santa Ana, CA) following the manufacturer’s instructions. All samples were crushed using a FastPrep^TM^ FP120 machine (MP Biomedicals) using Lysing Matrix A tubes at speed level 4, three times for 20 s each. The primers 515F (5′-CCTACGGGAGGCAGCAG-3′) and 907R (5′-TTACCGCGGCTGCTGGC-3′) were chosen to amplify the V4–V5 hypervariable region of the 16S rRNA gene. The 20 μl PCR reaction mix consisted of 10 ng of template DNA,4 μl of 5×FastPfu Buffer, 2 μl of dNTP mix (2.5 mM each), 0.4 μl of Fast Pfu Polymerase (2.5 units), 0.4 μl of 10 μM barcode primer 515F, 0.4 μl of 10 μM primer 907R, and 12.75 μl of double distilled water. After denaturing at 94 °C for 2 min, the amplification was carried out with 25 cycles of 30 s at 94 °C, 30 s at 55 °C, 45 s at 72 °C and a final extension step at 72 °C for 10 min. The PCR products were purified with the PCR Clean-Up^TM^ kit (MO BIO Labs, Solana Beach, CA, USA) and used as a template for direct sequencing.

### Sequencing using MiSeq PE300

Amplicons were sent out for pyrosequencing on the Illumina platform. The Majorbio-Shanghai. Illumina MiSeq PE300 was used to sequence the samples. After sequencing, the Trimmomatic^82^ was used to process the raw sequences, and the PE reads were overlapped by flash^83^ to assemble the final V4-V5 tag sequences. The uparse method (version 7.1) on the Usearch software platform (version 7.1 http://qiime.org/)^84^ was employed to assign operational taxonomic units (OTUs) to the 16S rRNA at a cutoff level of 3%. The Uchime (version 4.2.40)^85^ method was used to correctly detect and remove chimeras produced by PCR. To compare relative differences between the samples, a randomly selected subset of 41105 sequences per sample was performed for downstream analyses.

### Statistical analysis

An analysis of variance (ANOVA) was performed to evaluate the main effects of the agricultural system, farm location, crop type and the interactions of these factors on the soil physical and chemical analyzed. Based on the OTU results, the rarefaction curve and Shannon index curve were analyzed using Mothur (version v.1.30.1)^86^, and the richness estimators (Chao1, Abundance-based and Coverage Estimator (ACE)) and diversity indices (Shannon and Simpson) were determined using Mothur. Principal component analysis (PCA) and redundancy analysis (RDA) was performed with the rda() function in the vegan package in R (Version 3.0.2, vegan package)^87^. A Venn diagram was generated using R (Version 3.0.2, venn Diagram package). LEfSe^29^ was used to find indicator bacterial groups specialized within the organic and conventional types of samples. Statistical analysis was conducted using SPSS 13.0.

## Additional Information

**How to cite this article**: Wang, W. *et al*. Consistent responses of the microbial community structure to organic farming along the middle and lower reaches of the Yangtze River. *Sci. Rep*. **6**, 35046; doi: 10.1038/srep35046 (2016).

## Supplementary Material

Supplementary Information

## Figures and Tables

**Table 1:** Results of ANOVA showing the effects of management system, location and crop type in organic & conventional agriculture.

	TP	VP	pH	OM	SBD	TN	NO_3_-N	NH_4_-N	TK	VK
management system	***	**	***	***	ns	ns	***	***	***	***
Location	***	***	***	***	*	ns	***	***	***	***
crop type	ns	ns	ns	ns	ns	ns	ns	ns	ns	ns
management system *Location	***	***	***	***	*	ns	***	***	***	***
crop type *Location	ns	ns	ns	ns	ns	ns	ns	ns	ns	ns
management system *Location *crop type	ns	ns	ns	ns	ns	ns	ns	ns	ns	ns

(ns: not significant *p < 0.05. **p < 0.01. ***p < 0.001).

Management system: organic or conventional farming; Location: the sampling sites; crop type: tea vegetable and rice; TN: total nitrogen; TK: total K; TP: total P; VK: Available K; VP: Available P; NO_3_-N: nitrate nitrogen; NH_4_-N: ammonium nitrogen; OM: Organic matter; SBD: soil bulk density.

**Table 2:** Estimated OTU richness and diversity indices of the organic and conventional fields (P < 0.05, average value, n = 3, se = standard error).

Field	ACE		Chao1		Shannon		Simpson		OTU	
	Mean	se	Mean	se	Mean	se	Mean (×10^−3^)	se (×10^−3^)	Mean	se
OVL	4420.69a	104.95	4376.27a	121.18	6.59a	0.06	7.91b	1.97	3398.67a	62.07
CVL	4346.21a	105.14	4365.79a	97.97	5.94bc	0.47	31.24a	23.36	3180.33a	144.2
OVY	4378.75a	242.40	4471.81a	260.22	6.75a	0.20	3.42b	1.22	3372.67a	244.68
CVY	3072.43b	280.55	3133.69b	273.97	5.85c	0.21	13.77b	3.74	2285.33b	216.59
OPS	4420.24a	37.93	4418.45a	137.15	6.76a	0.07	3.06b	0.25	3323.00a	77.35
CPS	4455.93a	96.54	4474.06a	85.19	6.88a	0.05	3.34b	0.02	3522.00a	62.22
OPJ	4469.52a	12.98	4510.03a	75.43	6.68a	0.03	4.72b	0.34	3406.00a	21.93
CPJ	3224.41b	66.50	3242.03b	44.37	6.11bc	0.06	9.92b	1.04	2511.67b	68.71
OTC	2480.91c	86.39	2503.41c	123.33	6.20b	0.04	5.13b	0.34	2028.33c	48.01
CTC	1901.26d	93.61	1917.95d	111.96	5.49d	0.08	9.71b	0.78	1449.33d	60.93
OTW	2477.94c	64.46	2509.35c	57.17	5.98bc	0.14	7.87b	1.38	2016.33c	73.76
CTW	1885.52d	21.13	1883.49d	39.78	4.91e	0.14	46.60a	11.85	1492.00d	17.44

Note: Different letters in the same column indicates a significant difference (p < 0.05).
