# Supplementary material for: Consistent responses of the microbial community structure to organic farming along the middle and lower reaches of the Yangtze River
Source: Sci Rep. 2016 Oct 11;6:35046. doi: 10.1038/srep35046 (PMC5057158; doi:10.1038/srep35046)

**Title Page**

**Type of contribution:** regular papers

**Date of preparation:** 28 Juanury, 2016

**Number of text pages:** 28, **number of tables:** 2, **number of figures:** 4

**Number of Supplementary tables:** 5, **Number of Supplementary figures:** 2

**Title:** Consistent responses of the microbial community structure to organic farming along the middle and lower reaches of the Yangtze River

**Author names:** Wenhui Wang ^1, 2^, Hui Wang ^2^*, Youzhi Feng^2^, Lei Wang^4^, Xingji Xiao^4^, Yunguan Xi^4^, Xue Luo ^1^, Ruibo Sun^2^, Xianfeng Ye ^1^, Yan Huang ^1^, Zhengguang Zhang ^3^, Zhongli Cui ^1^*

**Affiliation:**

^1^Key Laboratory of Agricultural Environmental Microbiology of Ministry of Agriculture, Nanjing Agricultural University, Nanjing 210095, China

^2^Key Laboratory of Soil Environment and Pollution Remediation, Institute of Soil Science, Chinese Academy of Sciences, Nanjing 210008, China

^3^Department of Plant Pathology, College of Plant Protection, Nanjing Agricultural University, Nanjing 210095, China

^4^Nanjing Institute of Environmental Sciences, Ministry of Environmental Protection, Nanjing 210042, China

***Corresponding author**: Hui Wang

**Corresponding address:** Key Laboratory of Soil Environment and Pollution Remediation, Institute of Soil Science, Chinese Academy of Sciences, East Beijing Road 71, Nanjing 210008, China

**Tel.:** +862586881336, **Fax:** +8602586881000, **E-mail:** [hwang@issas.ac.cn](mailto:hychu@issas.ac.cn)

***Corresponding author**: Zhongli Cui

**Corresponding address:** Key Laboratory of Agricultural Environmental Microbiology of Ministry of Agriculture, Nanjing Agricultural University, Nanjing 210095, China

**Tel.:** +8602584396753, **Fax:** +8602584396753, **E-mail:** czl@njau.edu.cn

**Supplementary information I**

**Supplementary Figures and Tables**

**Table S1**

Table S1 Sampling points

| Field | Years in organic | Crop type | Soil type | Fertilizer input | Pesticide input |
| --- | --- | --- | --- | --- | --- |
| OVL | 12 | vegetable | Orthic Anthrosols | commercial organic fertilizer | none |
| CVL |  |  |  | 45% compound fertilizer | yes |
| OVY | 3 |  |  | organic fertilizer | none |
| CVY |  |  |  | 45% compound fertilizer | yes |
| OPS | 12 | rice | Stagnic Anthrosols | organic fertilizer | none |
| CPS |  |  |  | urea; compound fertilizer | yes |
| OPJ | 3 |  |  | organic fertilizer; rice bran | none |
| CPJ |  |  |  | 45% compound fertilizer; urea(0.3 t/hm^2^) | yes |
| OTC | 14 | tea | Perudic argosols | organic fertilizer | none |
| CTC |  |  |  | 45% compound fertilizer | yes |
| OTW | 18 |  | Udic argosols | organic fertilizer | none |
| CTW |  |  |  | compound fertilizer | yes |

The first letter of the field name represents the farming method; “O” represents organic farming; “C” represents conventional farming; the second letter represents the crop type; “V” represents vegetable; “P” represents paddy; “T” represents tea; the third letter represents the experimental region (the first letter of the sampling area)

**Table S2**

Table S2 Chemical properties of organic and conventional soils.

| Field | | TP（mg/kg） | | VP（mg/kg） | | pH | | OM（%） | | SBD | | TN（%） | | NO3-N（mg/kg） | | NH4-N（mg/kg） | | TK（%） | | VK（mg/kg） | |
| --- | --- | --- | --- | --- | --- | --- | --- | --- | --- | --- | --- | --- | --- | --- | --- | --- | --- | --- | --- | --- | --- |
|  |  | **O** | **C** | **O** | **C** | **O** | **C** | **O** | **C** | **O** | **C** | **O** | **C** | **O** | **C** | **O** | **C** | **O** | **C** | **O** | **C** |
| VL | Mean | 811.42a | 700.56b | 46.19a | 33.11b | 6.74b | 7.06a | 2.76a | 1.37b | 1.13 | 1.03 | 0.17 | 0.09 | 9.44b | 59.74a | 6.33 | 7.25 | 0.93a | 0.65b | 293.67a | 152.67b |
|  | se | 11.88 | 15.27 | 2.90 | 3.61 | 0.17 | 0.04 | 0.10 | 0.07 | 0.06 | 0.01 | 0.02 | 0.00 | 0.19 | 1.11 | 0.17 | 0.12 | 0.04 | 0.04 | 8.51 | 15.50 |
| VY | Mean | 937.64a | 765.67b | 56.26b | 120.28a | 7.5a | 5.13b | 1.84b | 2.03a | 1.2 | 1.30 | 0.08 | 0.13 | 4.19b | 5.9a | 4.36b | 46.73a | 0.87a | 0.79b | 250.50a | 158.17b |
|  | se | 13.04 | 24.66 | 1.04 | 7.85 | 0.10 | 0.13 | 0.03 | 0.10 | 0.05 | 0.14 | 0.00 | 0.01 | 0.03 | 0.16 | 0.09 | 1.03 | 0.04 | 0.01 | 7.40 | 10.28 |
| PS | Mean | 883.18a | 756.99b | 18.13 | 15.17 | 7.70a | 7.23b | 2.57a | 1.83b | 0.88b | 1.16a | 0.16 | 0.38 | 2.99b | 18.99a | 16.43a | 12.53b | 1.04a | 0.91b | 85.00 | 81.33 |
|  | se | 12.30 | 8.78 | 3.90 | 0.93 | 0.04 | 0.09 | 0.07 | 0.10 | 0.08 | 0.14 | 0.01 | 0.48 | 0.13 | 0.31 | 0.35 | 0.36 | 0.02 | 0.03 | 4.58 | 5.69 |
| PJ | Mean | 423.44b | 553.25a | 11.84b | 21.07a | 6.42a | 5.05b | 1.71b | 1.95a | 1.28a | 1.02b | 0.1 | 0.12 | 2.17b | 3.47a | 7.09b | 12.57a | 0.84a | 0.60b | 194.33a | 62.00b |
|  | se | 7.11 | 7.97 | 3.16 | 1.26 | 0.17 | 0.08 | 0.01 | 0.06 | 0.03 | 0.10 | 0.01 | 0.01 | 0.04 | 0.08 | 0.06 | 0.20 | 0.05 | 0.06 | 10.07 | 5.57 |
| TC | Mean | 490.57a | 167.89b | 157.88a | 4.11b | 3.89b | 4.32a | 5.08a | 1.04b | 1.20 | 1.22 | 0.26 | 0.04 | 94.59a | 13.05b | 48.27a | 11.83b | 1.66 | 1.67 | 150.67a | 70.00b |
|  | se | 7.40 | 2.27 | 13.28 | 1.90 | 0.06 | 0.11 | 0.03 | 0.05 | 0.29 | 0.13 | 0.01 | 0.01 | 1.71 | 0.10 | 1.67 | 0.43 | 0.02 | 0.02 | 7.77 | 6.56 |
| TW | Mean | 386.21b | 449.2a | 14.27b | 71.94a | 4.59a | 4.26b | 2.90 | 2.79 | 1.02 | 1.05 | 0.17 | 0.18 | 5.29b | 80.73a | 32.64b | 141.48a | 0.84a | 0.71b | 51.00 | 42.67 |
|  | se | 7.03 | 15.02 | 1.39 | 8.10 | 0.02 | 0.10 | 0.10 | 0.08 | 0.04 | 0.20 | 0.01 | 0.00 | 0.11 | 0.86 | 0.85 | 2.51 | 0.01 | 0.07 | 4.58 | 2.08 |
| Total | Mean | 655.41a | 565.59b | 50.76a | 44.28b | 6.14a | 5.51b | 2.81a | 1.84b | 1.12 | 1.13 | 0.16 | 0.16 | 19.78b | 30.31a | 19.19b | 38.73a | 1.03a | 0.89b | 170.86a | 94.47b |
|  | se | 233.79 | 217.29 | 52.43 | 41.51 | 1.47 | 1.24 | 1.14 | 0.57 | 0.17 | 0.16 | 0.06 | 0.20 | 34.51 | 30.15 | 16.64 | 49.19 | 0.30 | 0.38 | 88.46 | 46.51 |

Means of 3 replicates per treatment are presented (with standard deviation).

TN: total nitrogen; TK: total K; TP: total P; VK: Available K; VP: Available P；NO_3_–N: nitrate nitrogen; NH_4_–N: ammonium nitrogen; OM: Organic matter; SBD: soil bulk density; Different letters indicate significant differences among treatments (P < 0.05).

**Table S3** Sample list and sequencing information

| **field** | Sequences | Bases(bp) | Average Length(bp) |
| --- | --- | --- | --- |
| **OVL1** | 72214 | 28646703 | 396.69 |
| **OVL2** | 53949 | 21396496 | 396.61 |
| **OVL3** | 66861 | 26507072 | 396.45 |
| **CVL1** | 55667 | 22057042 | 396.23 |
| **CVL2** | 79300 | 31419551 | 396.21 |
| **CVL3** | 74792 | 29642961 | 396.34 |
| **OPS1** | 71348 | 28285750 | 396.45 |
| **OPS2** | 68505 | 27168753 | 396.60 |
| **OPS3** | 75877 | 30081224 | 396.45 |
| **CPS1** | 74889 | 29698388 | 396.57 |
| **CPS2** | 58856 | 23340003 | 396.56 |
| **CPS3** | 73993 | 29334379 | 396.45 |
| **OTC1** | 74772 | 29644881 | 396.47 |
| **OTC2** | 56999 | 22589283 | 396.31 |
| **OTC3** | 73511 | 29131162 | 396.28 |
| **CTC1** | 73725 | 29235569 | 396.55 |
| **CTC2** | 64424 | 25555247 | 396.67 |
| **CTC3** | 54960 | 21787599 | 396.43 |
| **OTW1** | 73995 | 29303986 | 396.03 |
| **OTW2** | 59500 | 23564433 | 396.04 |
| **OTW3** | 60103 | 23805929 | 396.09 |
| **CTW1** | 79174 | 31386757 | 396.43 |
| **CTW2** | 74120 | 29391764 | 396.54 |
| **CTW3** | 71630 | 28398825 | 396.47 |
| **OPJ1** | 52052 | 20652551 | 396.77 |
| **OPJ2** | 71939 | 28539600 | 396.72 |
| **OPJ3** | 60401 | 23966587 | 396.79 |
| **CPJ1** | 67331 | 26692252 | 396.43 |
| **CPJ2** | 75644 | 29991871 | 396.49 |
| **CPJ3** | 53187 | 21086838 | 396.47 |
| **OVY1** | 52682 | 20880199 | 396.34 |
| **OVY2** | 53299 | 21118764 | 396.23 |
| **OVY3** | 76775 | 30413124 | 396.13 |
| **CVY1** | 56698 | 22464569 | 396.21 |
| **CVY2** | 78446 | 31094681 | 396.38 |
| **CVY3** | 59911 | 23754376 | 396.49 |

**Table S4** An overwhelming majority of indicator microbial groups was found in the two types of fields

| Bacterial lineages in organic |  |
| --- | --- |
| Lacibacter | Bacteroidetes \ Sphingobacteriia |
| Niastella | Bacteroidetes \ Sphingobacteriia |
| Terrimonas | Bacteroidetes \ Sphingobacteriia |
| Ferruginibacter | Bacteroidetes \ Sphingobacteriia |
| Flavobacteria | Bacteroidetes \ Flavobacteriia |
| Flavobacteriales | Bacteroidetes \ Flavobacteriia |
| Flavobacteriaceae | Bacteroidetes \ Flavobacteriia |
| Flavobacterium | Bacteroidetes \ Flavobacteriia |
| Hamadaea | Actinobacteria \ Actinobacteridae |
| Catelliglobosispora | Actinobacteria \ Actinobacteridae |
| Luedemannella | Actinobacteria \ Actinobacteridae |
| Longispora | Actinobacteria \ Actinobacteridae |
| Kribbella | Actinobacteria \ Actinobacteridae |
| Streptosporangium | Actinobacteria \ Actinobacteridae |
| Pseudonocardia | Actinobacteria \ Actinobacteridae |
| Nocardiaceae | Actinobacteria \ Actinobacteridae |
| Phycisphaeraceae | Planctomycetes \ Phycisphaerae |
| Phycisphaera | Planctomycetes \ Phycisphaerae |
| Gemmata | Planctomycetes \ Planctomycetia |
| Planctomyces | Planctomycetes \ Planctomycetia |
| Zavarzinella | Planctomycetes \ Planctomycetia |
| Armatimonadetes_norank | Armatimonadetes |
| Chthonomonadetes | Armatimonadetes \ Chthonomonadetes |
| Chthonomonadales | Armatimonadetes \ Chthonomonadetes |
| Chthonomonadaceae | Armatimonadetes \ Chthonomonadetes |
| Chthonomonas | Armatimonadetes \Chthonomonadetes |
| Spartobacteria | Verrucomicrobia \ Spartobacteria |
| Chthoniobacterales | Verrucomicrobia \ Spartobacteria |
| Candidatus_Xiphinematobacter | Verrucomicrobia \ Spartobacteria |
| Xiphinematobacteraceae | Verrucomicrobia \ Spartobacteria |
| Erysipelotrichia | Firmicutes \ Erysipelotrichia |
| Erysipelotrichales | Firmicutes \ Erysipelotrichia |
| Erysipelotrichaceae | Firmicutes \ Erysipelotrichia |
| Planifilum | Firmicutes \ Bacilli |
| Elusimicrobia | Elusimicrobia |
| Elusimicrobia | Elusimicrobia \ Elusimicrobia |
| Gemmatimonadaceae_norank | Gemmatimonadetes \ Gemmatimonadales |
| Nordella | Proteobacteria\ Alphaproteobacteria |
| Bauldia | Proteobacteria\ Alphaproteobacteria |
| Beijerinckiaceae | Proteobacteria\ Alphaproteobacteria |
| Methylorosula | Proteobacteria\ Alphaproteobacteria |
| Rhodomicrobium | Proteobacteria\ Alphaproteobacteria |
| Pedomicrobium | Proteobacteria\ Alphaproteobacteria |
| Prosthecomicrobium | Proteobacteria\ Alphaproteobacteria |
| Labrys | Proteobacteria\ Alphaproteobacteria |
| Starkeya | Proteobacteria\ Alphaproteobacteria |
| Ensifer | Proteobacteria\ Alphaproteobacteria |
| Rhodopseudomonas | Proteobacteria\ Alphaproteobacteria |
| Mesorhizobium | Proteobacteria\ Alphaproteobacteria |
| Myxococcales | Proteobacteria\ Deltaproteobacteria |
| Phaselicystidaceae | Proteobacteria\ Deltaproteobacteria |
| Phaselicystis | Proteobacteria\ Deltaproteobacteria |
| Polyangiaceae | Proteobacteria\ Deltaproteobacteria |
| Sorangium | Proteobacteria\ Deltaproteobacteria |
| Byssovorax | Proteobacteria\ Deltaproteobacteria |
| Haliangiaceae | Proteobacteria\ Deltaproteobacteria |
| Haliangium | Proteobacteria\ Deltaproteobacteria |
| Myxococcus | Proteobacteria\ Deltaproteobacteria |
| Rhodospirillales_Family_Incertae_Sedis | Proteobacteria\ Alphaproteobacteria |
| Skermanella | Proteobacteria\ Alphaproteobacteria |
| Reyranella | Proteobacteria\ Alphaproteobacteria |
| Alteromonadales | Proteobacteria\ Gammaproteobacteria |
| Alteromonadaceae | Proteobacteria\ Gammaproteobacteria |
| Haliea | Proteobacteria\ Gammaproteobacteria |
| Desulfurellales | Proteobacteria\ Deltaproteobacteria |
| Desulfurellaceae | Proteobacteria\ Deltaproteobacteria |
| Thiotrichales | Proteobacteria\ Gammaproteobacteria |
| Thiotrichaceae | Proteobacteria\ Gammaproteobacteria |
| Cupriavidus | Proteobacteria\ Betaproteobacteria |
| Polaromonas | Proteobacteria\ Betaproteobacteria |
| Geminicoccus | Proteobacteria\ Alphaproteobacteria |
| Novosphingobium | Proteobacteria\ Alphaproteobacteria |
|  |  |
|  |  |
| Bacterial lineages in conventional |  |
| Proteobacteria | Proteobacteria |
| Gammaproteobacteria | Proteobacteria\ Gammaproteobacteria |
| Xanthomonadales | Proteobacteria\ Gammaproteobacteria |
| Xanthomonadaceae | Proteobacteria\ Gammaproteobacteria |
| Ottowia | Proteobacteria\ Betaproteobacteria |
| Schumannella | Actinobacteria \ Actinobacteridae |
| Sporichthyaceae | Actinobacteria \ Actinobacteridae |
| Nakamurella | Actinobacteria \ Actinobacteridae |
| Nakamurellaceae | Actinobacteria \ Actinobacteridae |
| Dermacoccaceae | Actinobacteria \ Actinobacteridae |
| Flexivirga | Actinobacteria \ Actinobacteridae |
| Amnibacterium | Actinobacteria \ Actinobacteridae |
| Sporobacter | Firmicutes\ Clostridia |
| Alicyclobacillus | Firmicutes \ Bacilli |
| Alkaliphilus | Firmicutes \ Clostridia |
| Ruminococcaceae_Incertae_Sedis | Firmicutes \ Clostridia |
| Ktedonobacteria_norank | Chloroflexi \ Ktedonobacteria |
| Sphaerobacteraceae | Chloroflexi \ Sphaerobacteridae |
| Sphaerobacter | Chloroflexi \ Sphaerobacteridae |
| Sphaerobacterales | Chloroflexi \ Sphaerobacteridae |
| Pullulanibacillus | Firmicutes \ Bacilli |
| Sporolactobacillaceae | Firmicutes \ Bacilli |
| Deinococcus_Thermus | Deinococcus-Thermus |
| Deinococci | Deinococcus-Thermus \ Deinococci |
| Acidiphilium | Proteobacteria\ Alphaproteobacteria |
| Terriglobus | Acidobacteria \ Acidobacteriales |

**Table S5**

Table S5 The significant environmental variables were identified by a forward selection using Monte Carlo permutations (999 permutations with a p-value).

|  | Paddy | | | Vegetable | | | Tea | | |
| --- | --- | --- | --- | --- | --- | --- | --- | --- | --- |
|  | r^2^ | Pr(>r) |  | r^2^ | Pr(>r) |  | r^2^ | Pr(>r) |  |
| TP | 0.586 | 0.023 | * | 0.093 | 0.644 |  | 0.553 | 0.024 | * |
| VP | 0.639 | 0.015 | * | 0.882 | 0.003 | ** | 0.158 | 0.462 |  |
| pH | 0.339 | 0.132 |  | 0.910 | 0.002 | ** | 0.285 | 0.231 |  |
| OM | 0.490 | 0.041 | * | 0.387 | 0.09 | . | 0.316 | 0.181 |  |
| SBD | 0.715 | 0.006 | ** | 0.415 | 0.093 | . | 0.174 | 0.458 |  |
| TN | 0.236 | 0.32 |  | 0.342 | 0.159 |  | 0.479 | 0.056 | . |
| NO_3_-N | 0.045 | 0.834 |  | 0.220 | 0.312 |  | 0.241 | 0.32 |  |
| NH_4_-N | 0.796 | 0.003 | ** | 0.983 | 0.001 | *** | 0.267 | 0.272 |  |
| TK | 0.246 | 0.262 |  | 0.262 | 0.248 |  | 0.414 | 0.112 |  |
| VK | 0.893 | 0.007 | ** | 0.508 | 0.02 | * | 0.015 | 0.935 |  |

Signif. codes: 0 ‘***’ 0.001 ‘**’ 0.01 ‘*’ 0.05 ‘.’ 0.1 ‘ ’ 1

P value based on 999 permutations.

The soil total N (TN), soil pH (pH), organic matter (OM), soil bulk density (SBD), soil total K (TK), available K (VK), total P (TP), available P (VP), soil nitrate nitrogen (NO_3_–N) and ammonium nitrogen (NH_4_–N).

**Figure captions**

**Figure S1.** Rarefaction curves for the OTUs number at 97% similarity - boxplot for every sample site (n=3). The first letter of the field name represents the farming method; “O” represents organic farming; “C” represents conventional farming; the second letter represents the crop type; “V” represents vegetable; “P” represents paddy; “T” represents tea; the third letter represents the experimental region (the first letter of the sampling area).

**Figure S2.** Redundancy analysis (RDA) diagram illustrating the relationship between the OTU-level community structure from different sampling sites and environmental variables. (A) Paddy soil (RDA1 and RDA3 were used to plot), (B) Vegetable soil, and (C) Tea soil.

**Figure S1.**


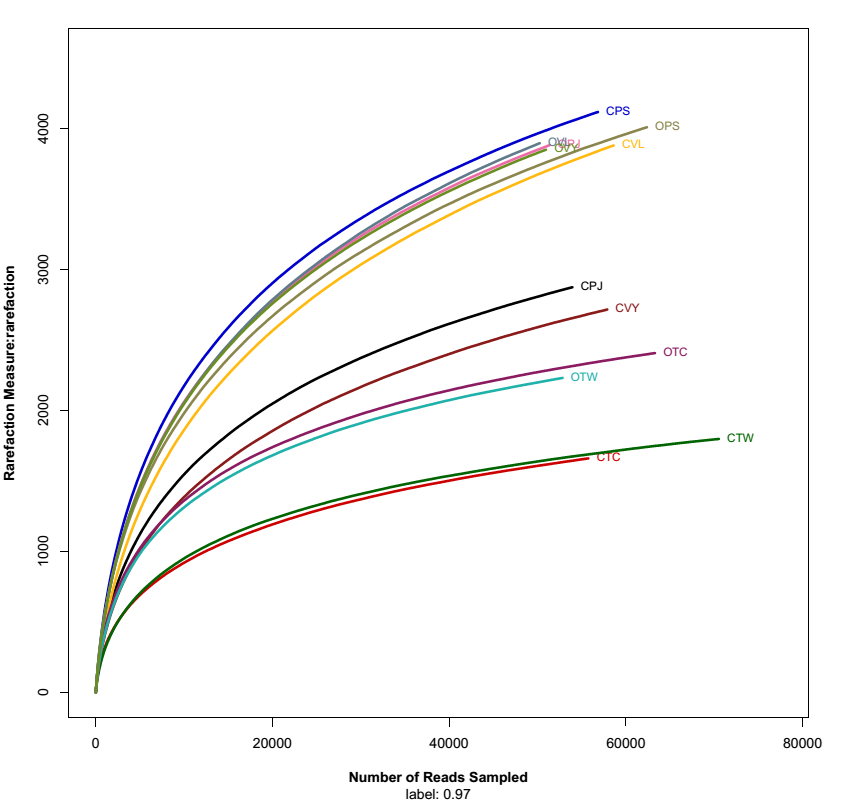


**Figure S2.**


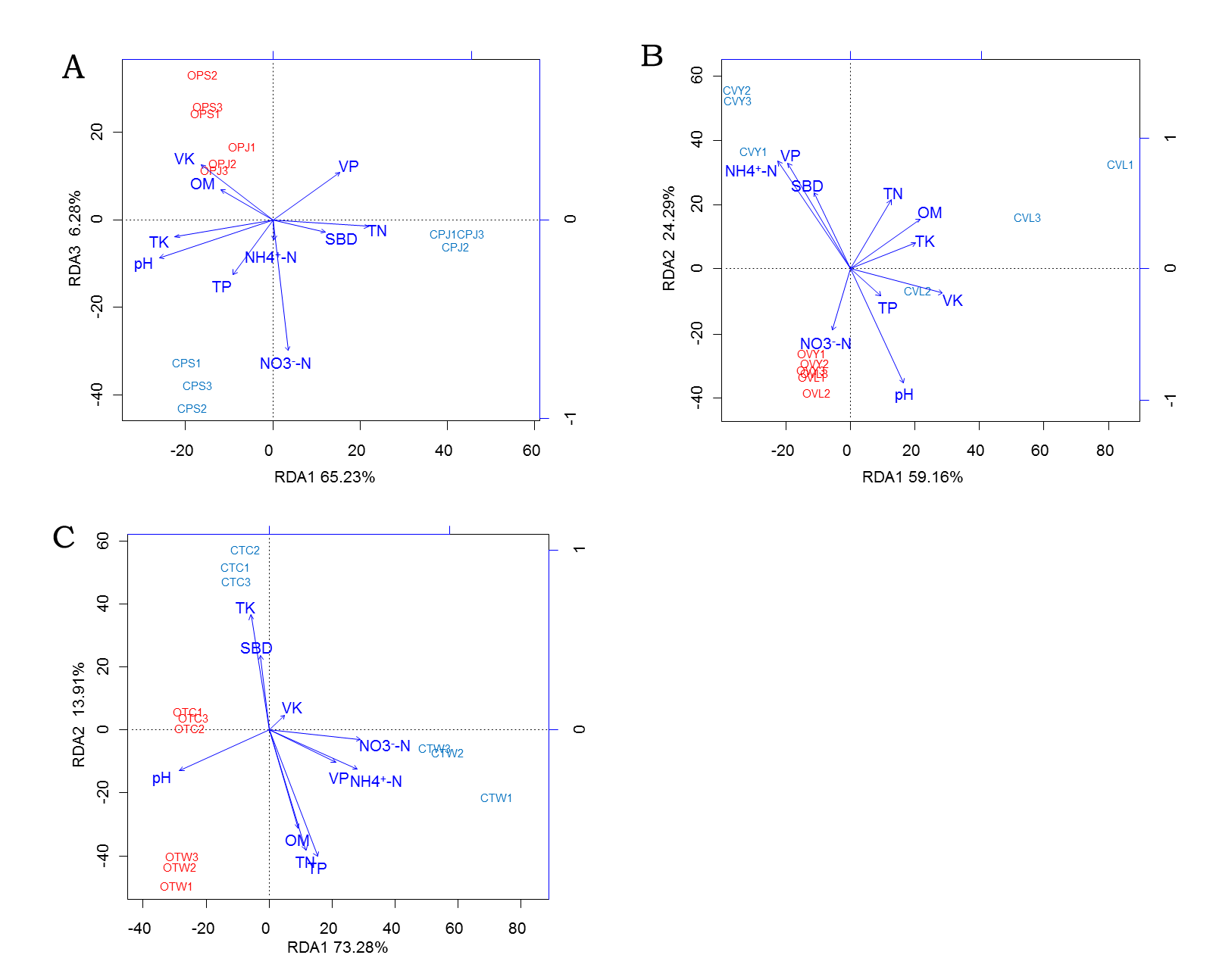

Supplement: Supplementary Information [file srep35046-s1.docx]
